# Gestational Diabetes and Genetics: *MTNR1B*, *CDKAL1*, and *IRS1* as Critical Players

**DOI:** 10.3390/genes17030287

**Published:** 2026-02-27

**Authors:** Guluzar Arzu Turan, Nehir Aran, Bulent Tolga Delibasi

**Affiliations:** 1 Department of Obstetrics and Gynecology, University of Colorado, Aurora, CO 80045, USA; 2 Department of Nutritional Sciences, University of Texas at Austin, Austin, TX 78705, USA; nehiraran@utexas.edu; 3 Long School of Medicine, University of Texas Health Science Center San Antonio, San Antonio, TX 78229, USA; delibasi@uthscsa.edu

**Keywords:** gestational diabetes, genetics, *MTNR1B*, *CDKAL1*, *IRS1*, genetic risk scores, β-cell function, polygenic risk score, precision prevention

## Abstract

Gestational diabetes mellitus (GDM) is a prevalent pregnancy complication with significant short- and long-term consequences for mothers and offspring. While environmental factors, such as obesity and diet, contribute to the risk, genetic predisposition also plays a role in the pathogenesis of GDM. Genome-wide association studies have identified multiple susceptibility loci, including *MTNR1B*, *CDKAL1*, and *IRS1*, which represent mechanistically distinct pathways affecting β-cell function, insulin secretion, and peripheral insulin signaling. This review provides a unified mechanistic framework explaining why these three genes, despite individually modest effect sizes, offer complementary insights into GDM pathophysiology that extend beyond other established loci such as *TCF7L2*. We critically evaluate the current evidence for genetic risk scores in GDM prediction, acknowledging that their incremental predictive value beyond traditional clinical factors remains modest AUC improvement typically <0.05). The integration of genetic variants with epigenetic modifications is discussed, with careful attention to distinguishing causal mechanisms from correlative findings. We emphasize significant limitations in current research, including population stratification, winner’s curse effects, and the predominance of East Asian cohorts. While genetic insights may eventually inform risk stratification, substantial barriers remain before clinical implementation, including insufficient predictive accuracy, lack of cost-effectiveness data, and limited generalizability across diverse populations. Future directions include integrating multi-omics data, developing ethnically validated polygenic risk scores, and conducting pragmatic randomized controlled trials to establish the clinical utility of precision prevention strategies.

## 1. Introduction

Gestational diabetes mellitus (GDM) is a common pregnancy complication characterized by impaired glucose tolerance due to inadequate pancreatic islet-cell compensation for pregnancy-induced insulin resistance. It affects nearly one in six pregnancies worldwide and poses significant risks for both mother and child, including macrosomia, preeclampsia, and future type 2 diabetes mellitus (T2DM) [1,2].

While environmental factors such as obesity and diet are well established as contributors, they do not fully explain the variability in GDM incidence. Increasing evidence suggests that genetic predisposition plays a contributory, though not deterministic, role in susceptibility [3].

**Figure 1:**
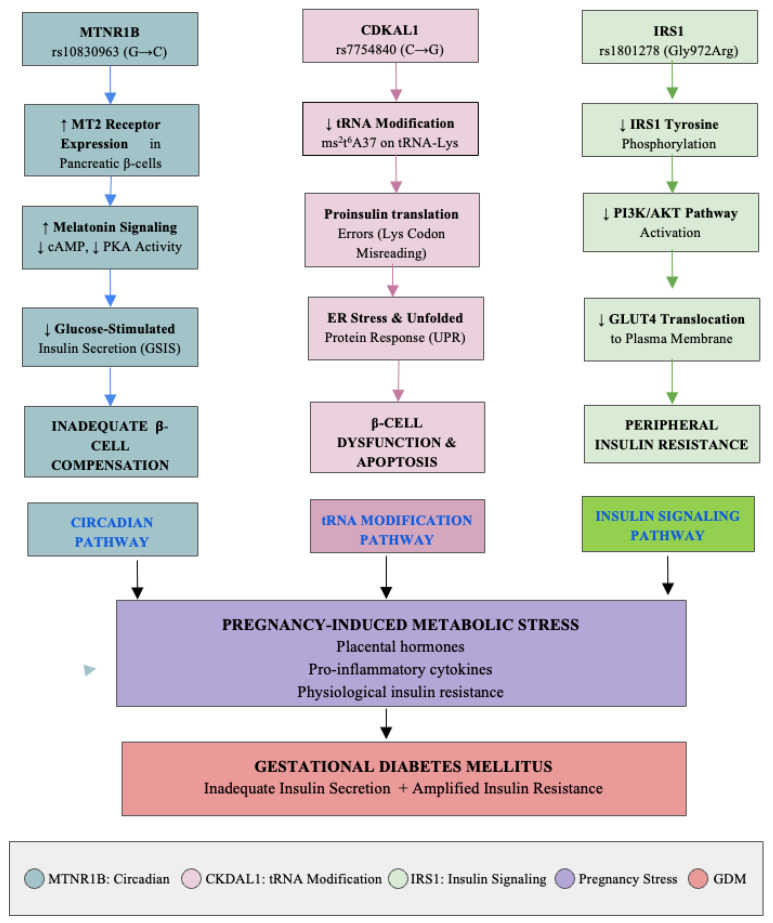
**Schematic representation of the three major genetic pathways contributing to gestational diabetes mellitus.** The *MTNR1B* pathway shows how the rs10830963 variant leads to MT2 receptor overexpression, melatonin-mediated inhibition of insulin secretion, and inadequate β-cell compensation. The rs10830963 G risk allele leads to overexpression of the MT2 melatonin receptor on pancreatic β-cells (upward arrow). Elevated MT2 signaling inhibits cAMP production via Gi protein coupling (inhibitory arrow), suppressing glucose-stimulated insulin secretion (GSIS; downward arrow on insulin granule release). The *CDKAL1* pathway illustrates how the rs7754840 variant causes reduced tRNA modification, proinsulin translation errors, ER stress, and β-cell dysfunction. The rs7754840 C risk allele impairs the methylthiotransferase enzyme that adds the ms2t6A37 modification to tRNA-Lys(UUU) (disrupted modification symbol). Hypomodified tRNA causes misreading of lysine codons during proinsulin translation (error symbol), producing misfolded proinsulin that accumulates in the ER, triggering the unfolded protein response (UPR), leading to β-cell dysfunction and apoptosis (cell damage symbol). The *IRS1* pathway demonstrates how the Gly972Arg variant impairs insulin receptor signaling, reduces PI3K/AKT activation, and decreases GLUT4 translocation. The Gly972Arg substitution impairs *IRS1* tyrosine phosphorylation (diminished phosphorylation symbol), reducing PI3K/AKT activation (downward arrows), impairing AS160 phosphorylation and GLUT4 translocation (impaired translocation arrow), and decreasing glucose uptake in muscle and adipose tissue. All three pathways converge during the third trimester, when pregnancy-induced metabolic stress and rising insulin resistance, driven by placental and hormonal factors such as hPL, TNF-α, cortisol, and progesterone, create a heightened demand for β-cell compensation. In this setting, combined defects in insulin secretion (associated with *MTNR1B* and *CDKAL1* variants) and impaired insulin signaling (linked to *IRS1*) limit the ability to maintain euglycemia, ultimately resulting in inadequate insulin secretion, increased insulin resistance, and the hyperglycemia characteristic of GDM.

Genome-wide association studies (GWAS) and candidate gene analyses have identified numerous loci associated with GDM, including *TCF7L2*, *MTNR1B*, *CDKAL1*, *KCNQ1*, *IGF2BP2*, *IRS1*, and *GCK*. Among these, *TCF7L2* represents one of the most potent common genetic risk factors for T2DM, with the rs7903146 variant consistently demonstrating odds ratios of approximately 1.35–1.42 across multiple populations [4,5,6]. However, this review focuses specifically on *MTNR1B*, *CDKAL1*, and *IRS1* for several mechanistic reasons: these three genes represent distinct and complementary pathophysiological pathways, circadian-mediated insulin secretion (*MTNR1B*), tRNA modification affecting proinsulin translation (*CDKAL1*), and peripheral insulin signaling (*IRS1*). Unlike *TCF7L2*, which broadly affects Wnt signaling, *MTNR1B*, *CDKAL1*, and *IRS1* provide a more tractable framework for understanding the specific islet-cell dysfunction and insulin resistance that characterize GDM [7,8]. Furthermore, *MTNR1B* shows a notably stronger effect in GDM than in T2DM (OR ~1.41–1.57 vs. 1.09), suggesting pregnancy-specific relevance [9,10]. The mechanistic pathways linking these three genes to GDM pathophysiology are illustrated in Figure 1.

### Search Strategy

A narrative literature review was conducted covering publications from January 2000 through December 2025. The following electronic databases were systematically searched: PubMed/MEDLINE, Embase, Cochrane Library, Web of Science, and Scopus. Inclusion criteria comprised peer-reviewed original research articles and meta-analyses published in English that investigated genetic associations between GDM and variants in *MTNR1B*, *CDKAL1*, or *IRS1*, or that evaluated polygenic risk scores for the prediction of GDM. Exclusion criteria included case reports, editorials, conference abstracts without full-text availability, studies focusing exclusively on type 2 diabetes without GDM-specific analyses, studies with fewer than 50 participants, and non-English-language publications. Additionally, the reference lists of all included articles and relevant review papers were manually screened to identify further eligible studies.

## 2. Gestational Diabetes

GDM is defined as carbohydrate intolerance of variable severity with onset or first recognition during pregnancy. Diagnosis typically relies on oral glucose tolerance testing (OGTT) performed between 24 and 28 weeks of gestation, according to criteria established by the International Association of Diabetes and Pregnancy Study Groups (IADPSG) or American Diabetes Association (ADA) [7]. It should be noted that diagnostic criteria vary internationally, contributing to heterogeneity in reported prevalence and potentially affecting the comparability of genetic association studies across populations [11].

Several clinical and demographic factors increase the risk of GDM, including increasing maternal age, obesity, family history of diabetes, previous GDM or macrosomic infant, and polycystic ovary syndrome. These factors interact with genetic predisposition [1,12]. Importantly, traditional clinical risk factors, particularly pre-pregnancy body mass index (BMI), age > 35 years, and prior GDM, account for most of the predictable risk, with genetic factors providing only modest incremental information [13].

The core mechanism of GDM involves inadequate pancreatic β-cell compensation for pregnancy-induced insulin resistance. Placental hormones contribute to insulin resistance, while pro-inflammatory cytokines impair IRS-1 phosphorylation and glucose transporter type 4 (GLUT4) translocation [3,7].

Maternal complications include increased risk of preeclampsia, cesarean delivery, and progression to T2DM (up to 50% within 10 years) [14]. Fetal complications include macrosomia and the long-term risk of obesity [15]. Intergenerational effects are significant, as epigenetic changes in offspring may predispose them to cardiometabolic disorders [16,17].

## 3. Genetic Architecture of GDM

GDM shares substantial genetic overlap with T2DM. GWAS have identified multiple susceptibility loci, including *TCF7L2*, *MTNR1B*, *CDKAL1*, *KCNQ1*, *IGF2BP2*, *IRS1*, and *GCK* [7,18]. Table 1 provides a comprehensive summary of the key genetic variants associated with GDM, including their effect sizes, risk allele frequencies, and functional impacts across different populations.

Polygenic risk scores (PRS) have been proposed to improve the prediction of GDM. However, critical evaluation reveals that the incremental predictive value is modest. Studies combining genetic variants with clinical factors typically report AUC improvements of only 0.03–0.07 compared to clinical factors alone [19,20,21]. For context, a model using only BMI, age, and family history achieves AUC values of 0.67–0.78, while adding genetic information increases this to approximately 0.70–0.77, a clinically marginal improvement [13,19]. Net reclassification improvement (NRI) analyses have recently been reported, with one study showing NRI of 0.258 (95% CI: 0.135–0.382) when adding PRS to clinical factors [22], though such analyses remain uncommon in the GDM genetic literature. Table 2 summarizes current genetic risk score studies and their predictive performance metrics.

This review focuses on *MTNR1B*, *CDKAL1*, and *IRS1* within a mechanistic framework that integrates three complementary pathways. While *TCF7L2* shows the largest effect size for T2DM, its mechanism involves Wnt/β-catenin signaling pathways that may be less specifically relevant to pregnancy [23]. Similarly, *KCNQ1* has shown inconsistent associations with GDM across populations [24].

Among the most studied genes, *MTNR1B* shows a notably effect size for GDM compared to T2DM (OR ~1.33–1.57, 95% CI: 1.21–1.70 vs. OR ~1.09, 95% CI: 1.06–1.12), followed by *CDKAL1* (OR ~1.36–1.52, 95% CI: 1.13–1.67) and *IRS1* (OR ~1.20–1.39, 95% CI: 1.08–1.79 under recessive model) [9,25,26]. Notably, the *IRS1* association was specific to the recessive model for rs1801278. Although these associations are statistically significant, their effect sizes correspond to modest absolute risk differences at the individual level. Therefore, the link between *IRS1* and GDM should be interpreted cautiously, as robust statistical significance has been observed only under the recessive genetic model.

**Table 2:** Summary of Genetic Risk Score Performance for GDM Prediction.

Study	Population	SNPs	AUC (PRS)	AUC (Clinical)	AUC (Combined)
Powe et al., 2018 [27]	European	8 T2DM	0.55–0.58	0.68–0.72	0.70–0.75 (+0.03)
Tam et al., 2025 [28]	East Asian	20–30	0.62–0.65	0.65–0.70	0.70–0.75 (+0.07)
Fang et al., 2022 [18]	Chinese	12 GDM	0.58–0.62	0.72–0.76	0.75–0.78 (+0.03)
Elliott et al., 2024 [29]	Multi-ethnic	GDM PRS	0.60–0.65	0.70–0.75	0.73–0.78 (+0.04)

*AUC = Area Under the Receiver Operating Characteristic Curve; PRS = Polygenic Risk Score; values in parentheses indicate incremental AUC improvement over clinical factors alone.*

### 3.1. Integration of Genetic and Epigenetic Mechanisms

Beyond genetic variants, epigenetic mechanisms have been associated with GDM. However, critical interpretation requires distinguishing between: (1) causal epigenetic changes that precede GDM, (2) changes that are consequences of hyperglycemia, and (3) correlative findings reflecting confounding [29,30]. It should be explicitly noted that, based on the currently available evidence, the directionality of the relationship between epigenetic modifications and GDM cannot be determined. Most existing studies are cross-sectional or case–control in design and therefore cannot establish whether observed epigenetic changes are causes, consequences, or merely correlates of gestational hyperglycemia. Prospective longitudinal studies with pre-conception or early-pregnancy epigenetic profiling are required to resolve this fundamental question of causality.

Most GDM epigenetic studies are cross-sectional, measuring methylation at or after diagnosis, thereby precluding the establishment of temporal precedence. Differential methylation of *KCNQ1*, *PTPRN2*, and *TXNIP* in maternal samples has been reported [9,31], but whether these changes cause or result from hyperglycemia remains unclear. The few longitudinal studies with pre-pregnancy samples suggest some methylation differences may precede GDM [32], but replication is limited.

Tissue specificity presents another challenge. Most human studies examine peripheral blood or placental tissues, which may not reflect methylation patterns in metabolically relevant tissues such as pancreatic islets [33]. Regarding intergenerational effects, differential methylation in offspring suggests that intrauterine hyperglycemia may influence future metabolic risk [16], but human evidence for true epigenetic transmission remains circumstantial [34].

### 3.2. MTNR1B: Linking Circadian Rhythm to Glucose Homeostasis

The *MTNR1B* gene encodes melatonin receptor 1B (MT2), involved in circadian rhythm regulation. Variants such as rs10830963 and rs1387153 are robust genetic markers for GDM [8]. GWAS have reported odds ratios up to 3.07 (95% CI: 1.54–6.11) in younger women (<30 years), though this estimate comes from a relatively small subgroup and should be interpreted cautiously [35]. More robust estimates from larger meta-analyses suggest ORs of 1.20–1.40 (95% CI: 1.10–1.55) [36]. The *MTNR1B* molecular pathway is illustrated in Figure 1.

Mechanistically, risk alleles have been associated with increased *MTNR1B* expression in pancreatic islets in vitro. However, the functional consequences in human islets in vivo remain incompletely characterized, and the relationship between genotype and islet expression has not been consistently replicated [37,38]. Regarding placental effects, one study reported elevated MT2 expression in GDM placentas; whether this directly enhances glucose transfer to the fetus and contributes to macrosomia remains to be investigated [39].

Meta-analyses confirm associations between *MTNR1B* variants and GDM risk, with pooled ORs ranging from 1.12 to 1.88 across genetic models and populations [40,41]. Notably, effect sizes appear stronger in Asian populations, though this may reflect publication bias or population-specific patterns of linkage disequilibrium rather than true biological differences.

Circadian rhythm disruptions, such as shift work and sleep deprivation, may increase the risk of GDM through several interrelated mechanisms. Shift workers experience misalignment between endogenous circadian rhythms and behavioral cycles, leading to altered melatonin secretion patterns that can impair glucose-stimulated insulin secretion [42]. Sleep deprivation, common during pregnancy, activates the hypothalamic–pituitary–adrenal axis, increases cortisol levels, and promotes insulin resistance in peripheral tissues [43,44]. Epidemiological studies have demonstrated that rotating night-shift work is associated with a 1.5- to 2-fold increase in the risk of diabetes, with the association being stronger among women who worked night shifts for longer durations [45]. In pregnancy specifically, evening shift work is associated with approximately 75% increased odds of GDM [46]. Furthermore, disrupted sleep architecture reduces slow-wave sleep, which is critical for optimal insulin sensitivity and islet cell function [45,47].

Gene–environment interactions may modify the functional impact of *MTNR1B* variants on GDM risk. The rs10830963 risk allele appears to have enhanced effects in individuals with disrupted circadian rhythms, suggesting that environmental factors can amplify genetic susceptibility. Studies have shown that carriers of the *MTNR1B* risk allele who consume meals late at night exhibit greater impairment in glucose tolerance compared to non-carriers [48,49]. These findings suggest that lifestyle modifications targeting circadian alignment could potentially attenuate genetic risk in *MTNR1B* variant carriers.

### 3.3. CDKAL1: Regulator of β-Cell Function and Insulin Biosynthesis

*CDKAL1*, located on chromosome 6p22.3, plays a critical role in tRNA modification for accurate proinsulin translation. The rs7754840 variant has been robustly associated with GDM and T2DM, with meta-analyses confirming significant associations across multiple genetic models [26,50]. While rs7756992 also shows a significant association with GDM, trial sequential analysis suggests the evidence is less definitive than for rs7754840, warranting further replication [26]. The *CDKAL1* pathway is depicted in Figure 1.

The *CDKAL1* gene encodes CDK5 regulatory subunit-associated protein 1-like 1, which functions as a methylthiotransferase essential for tRNA modification in pancreatic β-cells. Specifically, *CDKAL1* catalyzes the 2-methylthio (ms2) modification of N6-threonylcarbamoyladenosine (t6A) at position 37 of tRNA-Lys(UUU), producing ms2t6A37. This modification is critical for accurate codon-anticodon recognition during translation of proinsulin mRNA, which contains multiple lysine (AAA and AAG) codons [51]. When *CDKAL1* function is impaired, the resulting hypomodified tRNA leads to misreading of lysine codons during proinsulin translation, causing increased production of misfolded proinsulin that accumulates in the endoplasmic reticulum (ER). This triggers ER stress and activates the unfolded protein response, which, over time, contributes to islet-cell dysfunction and apoptosis [52]. Furthermore, *CDKAL1* deficiency impairs first-phase insulin secretion by reducing dense-core granule docking at the plasma membrane, compromising the rapid insulin response to glucose stimulation, which is essential for maintaining postprandial glucose homeostasis.

Meta-analyses report associations with pooled ORs of approximately 1.12–1.76 (95% CI: 1.05–2.26), with stronger effects in East Asian populations [28,53]. However, several methodological concerns warrant caution: many included studies had small sample sizes (*n* < 500), which increases susceptibility to the winner’s curse. Additionally, heterogeneity across studies suggests that a single pooled effect estimate may be misleading [50,54].

Beyond GDM, *CDKAL1* polymorphisms have been linked to adverse pregnancy outcomes. One study reported associations with low birth weight (OR = 19.80, 95% CI: 2.15–182) and macrosomia (OR = 2.40, 95% CI: 1.17–4.93) [55]. The extremely high OR for low birth weight, with a very wide confidence interval, is unusual and likely reflects the small sample size (207 pairs); replication is essential. Notably, larger studies examining fetal *CDKAL1* genotype have found more modest effects on birth weight, with meta-analyses reporting approximately 20 g lower birth weight per risk allele [56,57].

Notable differences in the strength of association between *CDKAL1* variants and GDM have been observed across populations. East Asian populations, particularly Chinese and Korean cohorts, have demonstrated strong associations (OR 1.30–1.52), with the Korean GWAS reporting the strongest effect (OR 1.518, *p* = 6.65 × 10^−16^) [24,28]. However, meta-analyses comparing East Asian and European populations for T2D suggest similar effect sizes across ancestries [58]. These differences may reflect several factors: variations in risk allele frequencies, distinct linkage disequilibrium patterns that may influence which functional variant is being tagged, and potential interactions with population-specific dietary patterns and metabolic phenotypes [59]. Additionally, data from African, Middle Eastern, and Hispanic populations remain limited, with some studies showing non-significant associations [60], highlighting a critical gap in our understanding of the global applicability of *CDKAL1*-based risk assessment.

### 3.4. IRS1: Insulin Signaling and Resistance

*IRS1* encodes a key adaptor protein in the PI3K/AKT signaling pathway. Variants such as rs1801278 (Gly972Arg) and rs2943641 have been associated with GDM susceptibility in multiple studies and meta-analyses [25,36,61,62]. The *IRS1* signaling pathway is shown in Figure 1.

*IRS1* polymorphisms affect insulin signaling through multiple mechanisms involving protein phosphorylation and downstream glucose transporter trafficking. The Gly972Arg variant (rs1801278) introduces an amino acid substitution that alters the protein’s ability to serve as a substrate for insulin receptor tyrosine kinase phosphorylation. Under normal conditions, insulin binding triggers *IRS1* tyrosine phosphorylation at multiple sites, creating docking sites for PI3K and activating the PI3K/AKT pathway. Studies in IRS-1-deficient mice demonstrated that IRS-1 plays central roles in insulin-induced glucose transport and protein synthesis, with IRS-1 deficiency resulting in a 70–80% reduction in PI3-kinase activity [63,64]. In patients with type 2 diabetes, skeletal muscle cells show reduced IRS-1 tyrosine phosphorylation and a more than twofold increase in inhibitory serine 636 phosphorylation [65]. This impaired phosphorylation cascade reduces AKT activation, thereby directly compromising GLUT4 translocation to the plasma membrane. AKT normally phosphorylates AS160 (TBC1D4), relieving its inhibitory effect on Rab GTPases that mediate GLUT4 vesicle fusion with the cell membrane. Consequently, *IRS1* variant carriers show reduced insulin-stimulated glucose uptake in skeletal muscle and adipose tissue. During pregnancy, as insulin sensitivity declines, functional deficits in *IRS1* signaling may become more pronounced, thereby impairing glucose clearance.

The evidence for *IRS1* variants in GDM is notably inconsistent, warranting detailed examination. Ustianowski et al. found no overall significant association between rs2943641 and GDM in a Polish cohort (*n* = 406), though individuals with the TT genotype exhibited higher fasting glucose (*p* < 0.05) [66]. In contrast, Zhang et al. reported an increased risk of GDM among Caucasians carrying the T allele of rs1801278. Several factors may explain these discrepancies, including the study of different single nucleotide polymorphisms (SNPs) (rs2943641 vs. rs1801278) with distinct functional consequences, population stratification (given that allele frequencies vary across ethnic groups), and small sample sizes, which lead to imprecise estimates and potential false positives [25]. Additionally, publication bias favoring positive findings may inflate meta-analytic estimates [67].

Meta-analyses report inconsistent associations for rs1801278. Shen et al. (2024), in an updated meta-analysis of 8 studies (2116 cases, 2661 controls), found no significant association under the dominant model (OR = 1.22, 95% CI: 0.88–1.70) or allele model (OR = 1.24, 95% CI: 0.91–1.68), though the recessive model showed a significant protective effect for the TT genotype (OR = 0.37, 95% CI: 0.16–0.86, *p* = 0.030) [68]. No publication bias was detected in this meta-analysis, although earlier meta-analyses have noted that some GDM genetic associations were significant only after excluding studies with publication bias [36]. The evidence for *IRS1* as a GDM susceptibility gene should be considered preliminary.

*IRS1* variants are considered to confer a lower risk for GDM compared to *MTNR1B* or *CDKAL1* for several mechanistic and epidemiological reasons. First, while *MTNR1B* and *CDKAL1* directly impair insulin secretion, a critical defect in GDM pathophysiology, *IRS1* affects peripheral insulin sensitivity, which represents a more distal and compensable aspect of glucose homeostasis. If insulin resistance is present, β-cells maintain normal glucose tolerance by increasing insulin output; only when β-cells cannot release sufficient insulin in the presence of insulin resistance do glucose concentrations [69]. Second, the *IRS1* Gly972Arg variant has a relatively low population frequency (3–7% heterozygote carriers in European populations), limiting its population-attributable risk. Third, redundancy in the insulin signaling pathway, including IRS2, a common element of peripheral insulin signaling that can partially compensate for *IRS1* deficiency, attenuates the functional impact of the *IRS1* variant [70,71]. Fourth, the effect of *IRS1* variants may be strongly modified by environmental factors such as obesity and physical activity, leading to inconsistent associations across study populations with different lifestyle characteristics. Finally, the meta-analytic effect sizes for *IRS1* (OR ~1.22–1.24, non-significant) are consistently smaller than those for *MTNR1B* (OR ~1.20–1.40) and *CDKAL1* (OR ~1.12–1.43), reflecting its lesser contribution to genetic risk.

### 3.5. Integrated Mechanistic Framework and Critical Assessment

Variants in *MTNR1B*, *CDKAL1*, and *IRS1* converge on two critical pathways in GDM pathophysiology: (1) Insulin secretion (*MTNR1B*, *CDKAL1*): *MTNR1B* affects circadian-mediated insulin release while *CDKAL1* impairs proinsulin translation fidelity, both reducing compensatory insulin output during pregnancy [59]; and (2) Insulin signaling (*IRS1*): Mutations disrupt PI3K/AKT signaling and GLUT4 translocation, exacerbating insulin resistance [70]. This framework explains why genetic variants with individually modest effects (OR 1.1–1.4) may act additively to increase GDM risk. Genetic risk score studies demonstrate this additive effect: compared with women in the lowest GRS tertile, those in the highest tertile had ORs of 2.72 (95% CI: 2.18–3.38) for GDM [6,18].

Critical evaluation of existing studies reveals several important limitations that temper enthusiasm for near-term clinical translation. First, the modest predictive accuracy of genetic risk scores represents a fundamental barrier; current PRS typically improve AUC by only 0.02–0.05 over clinical factors alone, an increment that is statistically significant but clinically marginal. For a screening test to change clinical management, substantially higher discrimination is typically required. Second, the lack of cost-effectiveness data is striking, despite numerous genetic association studies; no published analyses have formally evaluated whether genetic testing for GDM risk is cost-effective compared to current practice. Without such data, implementation decisions remain speculative. Third, limited generalizability across diverse populations is a major concern: approximately 60% of GDM genetic studies have been conducted in East Asian cohorts, with European populations comprising most of the remainder. African, Hispanic, South Asian, and other populations are severely underrepresented, yet GDM prevalence varies substantially across ethnic groups [72]. Fourth, the clinical utility of earlier intervention remains unproven, even if genetic testing could accurately identify high-risk women; evidence that earlier intervention improves long-term outcomes beyond standard GDM treatment is lacking [73]. Table 3 provides a comprehensive summary of these critical limitations and their implications for clinical translation.

## 4. Clinical Implications and Future Research Directions

Treatment of GDM at 24–28 weeks improves short-term pregnancy outcomes but may not fully address long-term risks. Women with GDM remain at substantially higher risk of developing T2DM (relative risk ~7-fold) [14]. A systematic review found no clear benefit of GDM treatment on long-term maternal diabetes risk or offspring outcomes [73], though this may reflect limitations in follow-up rather than a true absence of benefit.

Several mechanistic factors explain why therapeutic interventions for GDM primarily improve short-term rather than long-term health outcomes. First, current treatments, including dietary modification, exercise, and insulin or metformin, when necessary, are initiated at 24–28 weeks of gestation, after critical developmental windows have already occurred. By this point, fetal programming through epigenetic modifications in metabolically relevant tissues may already be established [75]. Second, while glucose control during pregnancy reduces acute complications such as macrosomia, it does not address the underlying genetic susceptibility or pre-existing metabolic dysfunction that predisposed the mother to GDM. The same β-cell defects and insulin resistance pathways that contributed to GDM persist postpartum and continue to increase the risk of T2DM. Third, interventions during pregnancy are necessarily time-limited and typically do not extend into the postpartum period, when lifestyle factors often deteriorate due to demands of infant care, sleep deprivation, and reduced physical activity [76]. Fourth, offspring exposed to intrauterine hyperglycemia, even when maternal glucose is subsequently controlled, may have already experienced alterations in appetite regulation, adipose tissue development, and pancreatic β-cell mass that predispose them to future metabolic disease. Finally, the inflammatory and oxidative stress pathways activated during GDM may cause persistent cellular damage that cannot be fully reversed by glycemic normalization during the remaining weeks of pregnancy.

### 4.1. Prevention Strategies: Evidence and Limitations

Evidence suggests GDM can sometimes be prevented through lifestyle interventions in high-risk women. The Finnish RADIEL trial demonstrated that early lifestyle counseling reduced the incidence of GDM [77]. A Cochrane meta-analysis confirmed that combined diet and exercise interventions reduce GDM risk by approximately 15% (RR 0.85, 95% CI: 0.71–1.01), though confidence intervals often cross unity [78].

### 4.2. Genetic Testing: Current Limitations and Future Potential

Genetic risk scores (GRS) combining multiple SNPs have been proposed for risk stratification. RADIEL trial demonstrated that lifestyle interventions are more effective in women with high genetic risk; the intervention reduced GDM only among those in the highest PRS tertile (OR 0.37, 95% CI 0.17–0.82) [79,80], though this requires confirmation in prospective trials. However, enthusiasm for ‘precision prevention’ in GDM should be tempered: current GRS achieve AUC values of approximately 0.60–0.65 alone, compared to 0.65–0.75 for clinical factors. Combined models reach 0.68–0.78, a modest improvement that shows limited utility in clinical identification of GDM cases [19,72,81].

### 4.3. Limitations of Current Genetic Studies

Current genetic research on GDM has several important limitations: Sample size and power, many studies include fewer than 1000 cases, and the largest GDM GWAS included approximately 12,332 cases [29], substantially smaller than T2DM studies. In initial discovery studies, the winner’s curse tends to overestimate effect sizes. Population stratification, a large proportion of studies have been conducted in East Asian populations, with implications for global generalizability [72]. Phenotype heterogeneityan GDM diagnostic criteria for GDM vary (IADPSG, Carpenter-Coustan, WHO 1999) [74], creating heterogeneity across studies. These limitations are summarized comprehensively in Table 3.

### 4.4. Future Research Priorities

To advance the field, future research should prioritize: large multi-ethnic GWAS with standardized GDM phenotyping; functional studies characterizing how genetic variants affect gene expression in metabolically relevant tissues; longitudinal epigenetic studies with pre-pregnancy samples to distinguish causal from consequential changes; prospective validation of GRS in diverse populations with pre-specified analyses of discrimination and NRI; randomized trials testing whether genotype-stratified interventions improve outcomes; and formal cost-effectiveness analyses comparing genetic testing strategies to current practice [9,16].

Epigenetic and microbiome research are emerging as important avenues for better understanding the consequences of GDM and developing novel interventions. Epigenetic studies can contribute by identifying specific DNA methylation patterns and histone modifications that mediate the intergenerational transmission of metabolic risk from mothers with GDM to their offspring. Such research may reveal biomarkers for early identification of at-risk pregnancies and potential therapeutic targets. Importantly, epigenetic marks are mechanistically reversible through enzymatic processes, unlike genetic variants, offering possibilities for pharmacological or nutritional interventions that could reprogram metabolic pathways [82,83]. Microbiome research offers complementary insights, as alterations in gut microbial composition have been associated with both GDM and T2DM. The maternal gut microbiome influences glucose metabolism through the production of short-chain fatty acids, modulation of intestinal permeability, and regulation of inflammatory pathways [84]. Furthermore, vertical transmission of microbiota from mother to infant may contribute to the metabolic programming of offspring. Integrating genetic, epigenetic, and microbiome data through multi-omics approaches could provide a more comprehensive understanding of GDM pathophysiology and identify novel prevention strategies [85].

Several future research directions may enhance the effectiveness of interventions to prevent the long-term consequences of GDM. First, preconception interventions targeting women at high genetic risk could address metabolic dysfunction before pregnancy, potentially preventing GDM altogether and reducing fetal programming effects. Second, the development of precision nutrition approaches that account for both genetic variants and microbiome composition may enable more effective dietary interventions tailored to individual metabolic profiles [86]. Third, extending postpartum follow-up and intervention programs beyond the traditional 6–12-week period could maintain beneficial lifestyle changes during the critical years when T2DM risk is highest. Fourth, investigating pharmacological agents that target specific pathways identified through genetic studies, such as melatonin receptor modulators for *MTNR1B* variant carriers, could enable genotype-guided prevention. Fifth, developing evidence-based guidelines for offspring surveillance and early intervention, informed by maternal GDM status and genetic risk, could interrupt the intergenerational cycle of metabolic disease. Finally, implementation science research is needed to translate genetic discoveries into accessible, equitable clinical tools that benefit diverse populations.

## 5. Conclusions

GDM is a complex condition influenced by environmental, clinical, and genetic factors. This review provides a mechanistic framework for understanding how *MTNR1B*, *CDKAL1*, and *IRS1*, which mediate circadian regulation of insulin secretion, proinsulin translation fidelity, and peripheral insulin signaling, respectively, contribute to GDM pathophysiology (Figure 1). While *MTNR1B* and *CDKAL1* show consistent and robust associations with GDM across multiple studies and genetic models, the evidence for *IRS1* is notably weaker and more inconsistent. *IRS1* is included in this framework for mechanistic completeness, as it represents the insulin signaling/resistance pathway, but its contribution to GDM genetic risk should be considered preliminary, pending further replication in larger, diverse cohorts.

However, enthusiasm for translating genetic findings into clinical practice should be tempered. Current genetic risk scores provide only modest incremental predictive value beyond traditional clinical factors (Table 2). Effect sizes for individual variants are small (OR typically 1.1–1.4), confidence intervals often overlap with unity, and substantial heterogeneity exists across populations (Table 1). Specifically, PRS alone achieves AUC values of 0.55–0.65, clinical factors alone achieve 0.65–0.76, and combined PRS + clinical models yield 0.70–0.78, representing an incremental improvement of only +0.03–0.07 (Table 2). This falls below the threshold typically considered clinically meaningful for changing screening practice. Based on these data, genetic testing and polygenic risk scores are not currently recommended for clinical use in GDM risk assessment. The evidence does not support incorporating PRS into routine prenatal screening due to: (1) insufficient discriminative accuracy (AUC improvement < 0.10); (2) absence of cost-effectiveness data; (3) limited validation in non-East Asian populations; and (4) no demonstrated improvement in clinical outcomes from genotype-guided interventions. Genetic approaches for GDM remain investigational tools requiring substantial further development before clinical adoption.

Epigenetic mechanisms represent an intriguing area of investigation, but current evidence cannot distinguish causal changes from consequences of hyperglycemia. Integration of genetic and epigenetic data into clinically useful risk models remains aspirational rather than achievable with current knowledge (Table 3).

Future research should prioritize large multi-ethnic cohorts, functional validation, and rigorous prospective evaluation of risk prediction models. Until such evidence accumulates, genetic testing for GDM risk should be considered a research tool rather than a clinical one.

**Figure 2:**
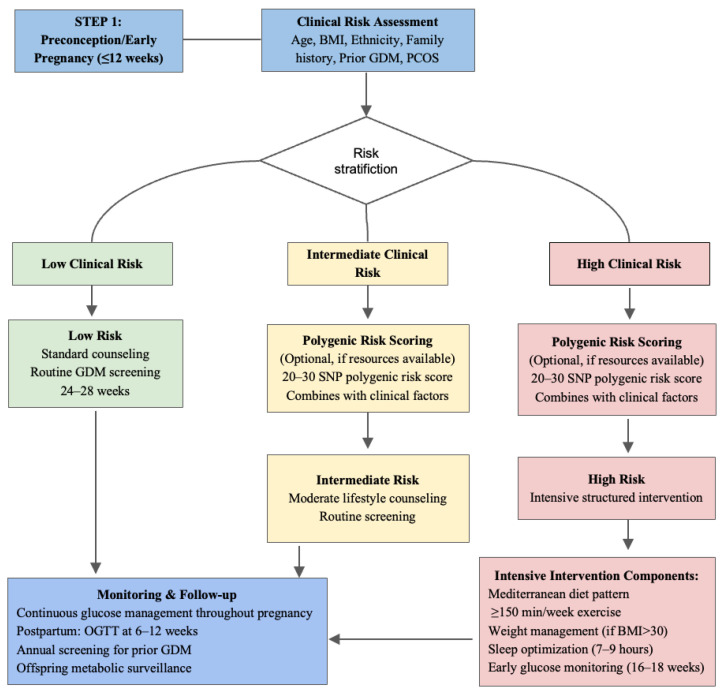
**Proposed clinical workflow for integrating genetic risk assessment into GDM screening and prevention strategies.** This algorithm illustrates how polygenic risk scoring could complement traditional clinical risk factors in a tiered approach to risk stratification, beginning in the preconception or early pregnancy period. This workflow is proposed and has NOT been validated as a clinical guideline. Evidence-supported steps: (1) standard clinical risk factor assessment (BMI, age, family history, prior GDM); established in ADA/IADPSG guidelines (2) OGTT at 24–28 weeks; standard of care (3) lifestyle intervention for high-risk women; RCT supported by RADIEL trial and Cochrane review. Proposed steps: (1) preconception PRS genotyping; not validated clinically (2) genotype-stratified risk thresholds; not established (3) pharmacogenomic-guided therapy (e.g., melatonin modulators for *MTNR1B* carriers); preclinical only (4) multi-omics integration; research stage.

In summary, while the genetic basis of GDM is increasingly well characterized, substantial barriers remain to the clinical implementation of genetic risk prediction. Key priorities for the field include: (1) conducting large, diverse GWAS to improve generalizability across populations; (2) performing rigorous functional studies to validate causal variants; (3) developing multi-omics approaches integrating genetic, epigenetic, transcriptomic, and metabolomic data; (4) conducting pragmatic randomized trials to evaluate clinical utility of genotype-stratified interventions; and (5) performing formal cost-effectiveness analyses to guide resource allocation. A proposed clinical integration framework (Figure 2) provides a template for future implementation, but realization of precision prevention in GDM will require sustained research investment and careful attention to equity across diverse populations.

## Figures and Tables

**Table 1:** Summary of Key Genetic Variants Associated with GDM.

Gene	Variant (rs ID)	Genetic Model and Odds Ratio	RAF EUR/EAS	Functional Impact
*MTNR1B*	rs10830963	Allelic: 1.41 (95% CI: 1.25–1.60)	0.30/0.45	↑MT2, ↓insulin secretion
*MTNR1B*	rs1387153	Allelic: 1.28 (1.20–1.37) Dominant: 1.41 (1.25–1.59) Recessive: 1.37 (~1.20–1.58) Homozygote: 1.64 (1.45–1.89)	0.28/0.42	linkage disequilibrium (LD) with rs10830963
*CDKAL1*	rs7754840	Allelic 1.33 (95% CI: 1.21–1.43)	0.28/0.38	↓proinsulin fidelity, ER stress
*CDKAL1*	rs7756992	Allelic 1.12 (95% CI: 1.05–1.20)	0.25/0.35	Inconsistent results
*IRS1*	rs1801278	Recessive 1.39 (95% CI: 1.08–1.79)	0.05/0.03	↓phosphatidylinositol 3-kinase (PI3K)/protein kinase B (AKT), ↓GLUT4
*IRS1*	rs2943641	Allelic 1.10 (95% CI: 1.04–1.17)	0.62/0.70	Regulatory effect

*RAF = Risk Allele Frequency; EUR = European; EAS = East Asian; LD = Linkage Disequilibrium.*

**Table 3:** Critical Limitations of Current GDM Genetic Research and Implications for Clinical Translation.

Limitation	Description	Clinical Implication
Small sample sizes	Most GWAS < 5000 cases; many candidate gene studies < 500	Imprecise effect estimates; susceptibility to false positives
Ethnic bias	~60% East Asian, ~30% European; African/Hispanic underrepresented	PRS may perform poorly in non-studied populations; health disparities risk
Winner’s curse	Discovery studies systematically overestimate true effect sizes	PRS based on inflated ORs will underperform in independent validation
Phenotype heterogeneity	Different diagnostic criteria (IADPSG, Carpenter-Coustan, World Health Organization (WHO)) [74]	Meta-analysis heterogeneity; difficulty comparing across studies
Modest predictive accuracy	PRS AUC 0.55–0.65 alone; +0.02–0.05 incremental improvement	Insufficient discrimination for clinical decision-making
No cost-effectiveness data	No formal economic analyses comparing genetic testing to standard care	Cannot justify resource allocation for genetic screening
Limited functional validation	Few CRISPR/mechanistic studies confirming causal variants	Uncertainty whether associated variants are truly causal

*GWAS = Genome-Wide Association Study; PRS = Polygenic Risk Score; OR = Odds Ratio; AUC = Area Under the Curve.*

## Data Availability

No new data were created or analyzed in this study.

## Abbreviations

**ADA**	American Diabetes Association
**AKT**	Protein kinase B
**AUC**	Area under the receiver operating characteristic curve
**BMI**	Body mass index
**cAMP**	Cyclic adenosine monophosphate
**CI**	Confidence interval
**DNA**	Deoxyribonucleic acid
**EAS**	East Asian
**ER**	Endoplasmic reticulum
**EUR**	European
**GDM**	Gestational diabetes mellitus
**GLUT4**	Glucose transporter type 4
**GRS**	Genetic risk score
**GSIS**	Glucose-stimulated insulin secretion
**GWAS**	Genome-wide association study/studies
**hPL**	Human placental lactogen
**IADPSG**	International Association of Diabetes and Pregnancy Study Groups
**LD**	Linkage disequilibrium
**mRNA**	Messenger ribonucleic acid
**ms2t6A37**	2-methylthio-N6-threonylcarbamoyladenosine at position 37
**NRI**	Net reclassification improvement
**OGTT**	Oral glucose tolerance test
**OR**	Odds ratio
**PI3K**	Phosphatidylinositol 3-kinase
**PRS**	Polygenic risk score
**RAF**	Risk allele frequency
**RCT**	Randomized controlled trial
**RR**	Relative risk
**SNP**	Single nucleotide polymorphism
**T2DM**	Type 2 diabetes mellitus
**TNF-α**	Tumor necrosis factor alpha
**tRNA**	Transfer ribonucleic acid
**UPR**	Unfolded protein response
**WHO**	World Health Organization

## References

[1] Sweeting A., Wong J., Murphy H.R., Ross G.P. (2022). A Clinical Update on Gestational Diabetes Mellitus. Endocr. Rev..

[2] Sweeting A., Hannah W., Backman H., Catalano P., Feghali M., Herman W.H., Hivert M.-F., Immanuel J., Meek C., Oppermann M.L. (2024). Epidemiology and management of gestational diabetes. Lancet.

[3] Ray G.W., Zeng Q., Kusi P., Zhang H., Shao T., Yang T., Wei Y., Li M., Che X., Guo R. (2024). Genetic and inflammatory factors underlying gestational diabetes mellitus: A review. Front. Endocrinol..

[4] Mahajan A., Taliun D., Thurner M., Robertson N.R., Torres J.M., Rayner N.W., Payne A.J., Steinthorsdottir V., Scott R.A., Grarup N. (2018). Fine-mapping type 2 diabetes loci to single-variant resolution using high-density imputation and islet-specific epigenome maps. Nat. Genet..

[5] Saxena R., Gianniny L., Burtt N.P., Lyssenko V., Giuducci C., Sjören M., Florez J.C., Almgren P., Isomaa B., Orho-Melander M. (2006). Common single nucleotide polymorphisms in *TCF7L2* are reproducibly associated with type 2 diabetes and reduce the insulin response to glucose in nondiabetic individuals. Diabetes.

[6] Ding W., Xu L., Zhang L., Han Z., Jiang Q., Wang Z., Jin S. (2018). Meta-analysis of association between *TCF7L2* polymorphism rs7903146 and type 2 diabetes mellitus. BMC Med. Genet..

[7] Mittal R., Prasad K., Lemos J.R.N., Arevalo G., Hirani K. (2025). Unveiling Gestational Diabetes: An Overview of Pathophysiology and Management. Int. J. Mol. Sci..

[8] Pervjakova N., Moen G.-H., Borges M.-C., Ferreira T., Cook J.P., Allard C., Beaumont R.N., Canouil M., Hatem G., Heiskala A. (2022). Multi-ancestry genome-wide association study of gestational diabetes mellitus highlights genetic links with type 2 diabetes. Hum. Mol. Genet..

[9] Lowe W.L. (2023). Genetics and Epigenetics: Implications for the Life Course of Gestational Diabetes. Int. J. Mol. Sci..

[10] Zhen J., Gu Y., Wang P., Wang W., Bian S., Huang S., Liang H., Huang M., Yu Y., Chen Q. (2024). Genome-wide association and Mendelian randomisation analysis among 30,699 Chinese pregnant women identifies novel genetic and molecular risk factors for gestational diabetes and glycaemic traits. Diabetologia.

[11] McIntyre H.D., Catalano P., Zhang C., Desoye G., Mathiesen E.R., Damm P. (2019). Gestational diabetes mellitus. Nat. Rev. Dis. Primers.

[12] Hughes Z.H., Hughes L.M., Khan S.S. (2023). Genetic contributions to risk of adverse pregnancy outcomes. Curr. Cardiovasc. Risk Rep..

[13] Ruiter M.L.-D., Kwee A., Naaktgeboren C.A., de Groot I., Evers I.M., Groenendaal F., Hering Y.R., Huisjes A.J.M., Kirpestein C., Monincx W.M. (2016). External validation of prognostic models to predict risk of gestational diabetes mellitus in one Dutch cohort: Prospective multicentre cohort study. BMJ.

[14] Bellamy L., Casas J.P., Hingorani A.D., Williams D. (2009). Type 2 diabetes mellitus after gestational diabetes: A systematic review and meta-analysis. Lancet.

[15] Ye W., Luo C., Huang J., Li C., Liu Z., Liu F. (2022). Gestational diabetes mellitus and adverse pregnancy outcomes: Systematic review and meta-analysis. BMJ.

[16] Kong D., Kowalik O., Garratt E., Godfrey K.M., Chan S.-Y., Teo A.K.K. (2025). Genetics and epigenetics in gestational diabetes contributing to type 2 diabetes. Trends Endocrinol. Metab..

[17] Al Bekai E., El Beaini C., Kalout K., Safieddine O., Semaan S., Sahyoun F., Ghadieh H.E., Azar S., Kanaan A., Harb F. (2025). The Hidden Impact of Gestational Diabetes: Unveiling Offspring Complications and Long-Term Effects. Life.

[18] Fang X., Jin L., Tang M., Lu W., Lai S., Zhang R., Zhang H., Jiang F., Luo M., Hu C. (2022). Common single-nucleotide polymorphisms combined with a genetic risk score provide new insights regarding the etiology of gestational diabetes mellitus. Diabet. Med..

[19] Kawai V.K., Levinson R.T., Adefurin A., Kurnik D., Collier S.P., Conway D., Stein C.M. (2017). A genetic risk score that includes common type 2 diabetes risk variants is associated with gestational diabetes. Clin. Endocrinol..

[20] Dziedziejko V., Safranow K., Tarnowski M., Pawlik A. (2019). Common Type 2 Diabetes Genetic Risk Variants Improve the Prediction of Gestational Diabetes. Horm. Metab. Res..

[21] Li Y., Yang M., Yuan L., Li T., Zhong X., Guo Y. (2023). Associations between a polygenic risk score and the risk of gestational diabetes mellitus in a Chinese population: A case-control study. Endocr. J..

[22] Cheng J., Meng C., Li J., Kong Z., Zhou A. (2024). Integrating polygenic risk scores in the prediction of gestational diabetes risk in China. Front. Endocrinol..

[23] Lyssenko V., Lupi R., Marchetti P., Del Guerra S., Orho-Melander M., Almgren P., Sjögren M., Ling C., Eriksson K.-F., Lethagen A.-L. (2007). Mechanisms by which common variants in the *TCF7L2* gene increase risk of type 2 diabetes. J. Clin. Invest..

[24] Kwak S.H., Kim S.-H., Cho Y.M., Go M.J., Cho Y.S., Choi S.H., Moon M.K., Jung H.S., Shin H.D., Kang H.M. (2012). A genome-wide association study of gestational diabetes mellitus in Korean women. Diabetes.

[25] Zhang C., Bao W., Rong Y., Yang H., Bowers K., Yeung E., Kiely M. (2013). Genetic variants and the risk of gestational diabetes mellitus: A systematic review. Hum. Reprod. Update.

[26] Yu X.Y., Song L.P., Wei S.D., Wen X.L., Liu D.B. (2021). CDK5 Regulatory Subunit-Associated Protein 1-Like 1 Gene Polymorphisms and Gestational Diabetes Mellitus Risk: A Trial Sequential Meta-Analysis of 13,306 Subjects. Front. Endocrinol..

[27] Powe C.E., Nodzenski M., Talbot O., Allard C., Briggs C., Leya M.V., Perron P., Bouchard L., Florez J.C., Scholtens D.M. (2018). Genetic Determinants of Glycemic Traits and the Risk of Gestational Diabetes Mellitus. Diabetes.

[28] Tam C.H.-T., Wang Y., Wang C.C., Yuen L.Y., Lim C.K.-P., Leng J., Wu L., Ng A.C.-W., Hou Y., Tsoi K.Y. (2025). Identification and Potential Clinical Utility of Common Genetic Variants in Gestational Diabetes among Chinese Pregnant Women. Diabetes Metab. J..

[29] Elliott A., Walters R.K., Pirinen M., Kurki M., Junna N., Goldstein J.I., Reeve M.P., Lemmelä S.M., Turley P., Lahtela E. (2024). Distinct and shared genetic architectures of gestational diabetes mellitus and type 2 diabetes. Nat. Genet..

[30] Sharp G.C., Salas L.A., Monnereau C., Allard C., Yousefi P., Everson T.M., Bohlin J., Xu Z., Huang R.-C., Reese S.E. (2017). Maternal BMI at the start of pregnancy and offspring epigenome-wide DNA methylation: Findings from the pregnancy and childhood epigenetics (PACE) consortium. Hum. Mol. Genet..

[31] Elliott H.R., Sharp G.C., Relton C.L., Lawlor D.A. (2019). Epigenetics and gestational diabetes: A review of epigenetic epidemiology studies and their use to explore epigenetic mediation and improve prediction. Diabetologia.

[32] Hivert M.-F., Cardenas A., Allard C., Doyon M., Powe C.E., Catalano P.M., Perron P., Bouchard L. (2020). Interplay of Placental DNA Methylation and Maternal Insulin Sensitivity in Pregnancy. Diabetes.

[33] Cardenas A., Gagné-Ouellet V., Allard C., Brisson D., Perron P., Bouchard L., Hivert M.-F. (2018). Placental DNA Methylation Adaptation to Maternal Glycemic Response in Pregnancy. Diabetes.

[34] Aiken C.E., Tarry-Adkins J.L., Ozanne S.E. (2016). Transgenerational effects of maternal diet on metabolic and reproductive ageing. Mamm. Genome.

[35] Zeng Q., Liu X., Liu J., Wu Y., Zhang Y., He Z., Wei Y., Zeng G., Zou D., Guo R. (2025). *MTNR1B* variants increase gestational diabetes mellitus risk in young Chinese pregnant women. Sci. Rep..

[36] Wu L., Cui L., Tam W.H., Ma R.C., Wang C.C. (2016). Genetic variants associated with gestational diabetes mellitus: A meta-analysis and subgroup analysis. Sci. Rep..

[37] Tuomi T., Nagorny C.L.F., Singh P., Bennet H., Yu Q., Alenkvist I., Isomaa B., Östman B., Söderström J., Pesonen A.-K. (2016). Increased Melatonin Signaling Is a Risk Factor for Type 2 Diabetes. Cell Metab..

[38] Singh T., Kalamajski S., Cunha J.P.M.C.M., Hladkou S., Roberts F., Gheibi S., Soltanian A., Farahmand K.Y., Ekström O., Mamidi A. (2025). Modeling Genetic Risk of beta-Cell Dysfunction in Human Induced Pluripotent Stem Cells From Patients Carrying the *MTNR1B* Risk Variant. J. Pineal Res..

[39] Wei L., Jiang Y., Gao P., Zhang J., Zhou X., Zhu S., Chen Y., Zhang H., Du Y., Fang C. (2023). Role of melatonin receptor 1B gene polymorphism and its effect on the regulation of glucose transport in gestational diabetes mellitus. J. Zhejiang Univ. Sci. B.

[40] Bai Y., Tang L., Li L., Li L. (2020). The roles of *ADIPOQ* rs266729 and *MTNR1B* rs10830963 polymorphisms in patients with gestational diabetes mellitus: A meta-analysis. Gene.

[41] Shan D., Wang A., Yi K. (2023). *MTNR1B* rs1387153 Polymorphism and Risk of Gestational Diabetes Mellitus: Meta-Analysis and Trial Sequential Analysis. Public. Health Genom..

[42] Qian J., Dalla Man C., Morris C.J., Cobelli C., Scheer F. (2018). Differential effects of the circadian system and circadian misalignment on insulin sensitivity and insulin secretion in humans. Diabetes Obes. Metab..

[43] Reutrakul S., Zaidi N., Wroblewski K., Kay H.H., Ismail M., Ehrmann D.A., Van Cauter E. (2011). Sleep disturbances and their relationship to glucose tolerance in pregnancy. Diabetes Care.

[44] Leproult R., Holmback U., Van Cauter E. (2014). Circadian misalignment augments markers of insulin resistance and inflammation, independently of sleep loss. Diabetes.

[45] Pan A., Schernhammer E.S., Sun Q., Hu F.B. (2011). Rotating night shift work and risk of type 2 diabetes: Two prospective cohort studies in women. PLoS Med..

[46] Wallace D.A., Reid K., Grobman W.A., Facco F.L., Silver R.M., Pien G.W., Louis J., Zee P.C., Redline S., Sofer T. (2023). Associations between evening shift work, irregular sleep timing, and gestational diabetes in the Nulliparous Pregnancy Outcomes Study: Monitoring Mothers-to-be (nuMoM2b). Sleep.

[47] Tasali E., Leproult R., Ehrmann D.A., Van Cauter E. (2008). Slow-wave sleep and the risk of type 2 diabetes in humans. Proc. Natl. Acad. Sci. USA.

[48] Lopez-Minguez J., Saxena R., Bandin C., Scheer F.A., Garaulet M. (2018). Late dinner impairs glucose tolerance in *MTNR1B* risk allele carriers: A randomized, cross-over study. Clin. Nutr..

[49] Garaulet M., Lopez-Minguez J., Dashti H.S., Vetter C., Hernández-Martínez A.M., Pérez-Ayala M., Baraza J.C., Wang W., Florez J.C., Scheer F.A. (2022). Interplay of Dinner Timing and *MTNR1B* Type 2 Diabetes Risk Variant on Glucose Tolerance and Insulin Secretion: A Randomized Crossover Trial. Diabetes Care.

[50] Guo F., Long W., Zhou W., Zhang B., Liu J., Yu B. (2018). *FTO*, *GCKR*, *CDKAL1* and *CDKN2A/B* gene polymorphisms and the risk of gestational diabetes mellitus: A meta-analysis. Arch. Gynecol. Obstet..

[51] Wei F.Y., Suzuki T., Watanabe S., Kimura S., Kaitsuka T., Fujimura A., Matsui H., Atta M., Michiue H., Fontecave M. (2011). Deficit of tRNA(Lys) modification by Cdkal1 causes the development of type 2 diabetes in mice. J. Clin. Invest..

[52] Ohara-Imaizumi M., Yoshida M., Aoyagi K., Saito T., Okamura T., Takenaka H., Akimoto Y., Nakamichi Y., Takanashi-Yanobu R., Nishiwaki C. (2010). Deletion of *CDKAL1* affects mitochondrial ATP generation and first-phase insulin exocytosis. PLoS ONE.

[53] Mao H., Li Q., Gao S. (2012). Meta-analysis of the relationship between common type 2 diabetes risk gene variants with gestational diabetes mellitus. PLoS ONE.

[54] Powe C.E., Kwak S.H. (2020). Genetic Studies of Gestational Diabetes and Glucose Metabolism in Pregnancy. Curr. Diab Rep..

[55] Yue S., Su M., Zhang Z., Li J., Leng J., Li W., Liu J., Zhang T., Qiao Y., Yu Z. (2025). Associations of maternal cyclin-dependent kinase 5 regulatory subunit-associated protein 1-like 1(*CDKAL1*) gene variants with adverse pregnancy outcome in Chinese women. BMC Pregnancy Childbirth.

[56] Andersson E.A., Pilgaard K., Pisinger C., Harder M.N., Grarup N., Færch K., Poulsen P., Witte D.R., Jørgensen T., Vaag A. (2010). Type 2 diabetes risk alleles near *ADCY5*, *CDKAL1* and *HHEX-IDE* are associated with reduced birthweight. Diabetologia.

[57] Freathy R.M., Bennett A.J., Ring S.M., Shields B., Groves C.J., Timpson N.J., Weedon M.N., Zeggini E., Lindgren C.M., Lango H. (2009). Type 2 diabetes risk alleles are associated with reduced size at birth. Diabetes.

[58] Tan J.T., Ng D.P.K., Nurbaya S., Ye S., Lim X.L., Leong H., Seet L.T., Siew W.F., Kon W., Wong T.Y. (2010). Polymorphisms identified through genome-wide association studies and their associations with type 2 diabetes in Chinese, Malays, and Asian-Indians in Singapore. J. Clin. Endocrinol. Metab..

[59] Hryniewicka J., Buczynska-Backiel A., Zbucka-Kretowska M., Kretowski A.J., Szelachowska M. (2026). Molecular Genetics of beta-Cell Compensation in Gestational Diabetes Mellitus: Insights from CDKAL1, SLC30A8 and HHEX. Int. J. Mol. Sci..

[60] Noury A.E., Azmy O., Alsharnoubi J., Salama S., Okasha A., Gouda W. (2018). Variants of *CDKAL1* rs7754840 (G/C) and *CDKN2A/2B* rs10811661 (C/T) with gestational diabetes: Insignificant association. BMC Res. Notes.

[61] Sami A., Javed A., Ozsahin D.U., Ozsahin I., Muhammad K., Waheed Y. (2025). Genetics of diabetes and its complications: A comprehensive review. Diabetol. Metab. Syndr..

[62] Prasad R.B., Kristensen K., Katsarou A., Shaat N. (2021). Association of single nucleotide polymorphisms with insulin secretion, insulin sensitivity, and diabetes in women with a history of gestational diabetes mellitus. BMC Med. Genom..

[63] Araki E., Lipes M.A., Patti M.-E., Brüning J.C., Haag B.H., Johnson R., Kahn C.R. (1994). Alternative pathway of insulin signalling in mice with targeted disruption of the IRS-1 gene. Nature.

[64] Yamauchi T., Tobe K., Tamemoto H., Ueki K., Kaburagi Y., Yamamoto-Honda R., Takahashi Y., Yoshizawa F., Aizawa S., Akanuma Y. (1996). Insulin signalling and insulin actions in the muscles and livers of insulin-resistant, insulin receptor substrate 1-deficient mice. Mol. Cell Biol..

[65] Bouzakri K., Roques M., Gual P., Espinosa S., Guebre-Egziabher F., Riou J.-P., Laville M., Le Marchand-Brustel Y., Tanti J.-F., Vidal H. (2003). Reduced activation of phosphatidylinositol-3 kinase and increased serine 636 phosphorylation of insulin receptor substrate-1 in primary culture of skeletal muscle cells from patients with type 2 diabetes. Diabetes.

[66] Ustianowski P., Malinowski D., Czerewaty M., Safranow K., Tarnowski M., Dziedziejko V., Pawlik A. (2022). *COBLL1* and *IRS1* Gene Polymorphisms and Placental Expression in Women with Gestational Diabetes. Biomedicines.

[67] Ioannidis J.P., Thomas G., Daly M.J. (2009). Validating, augmenting and refining genome-wide association signals. Nat. Rev. Genet..

[68] Shen L., Liu J., Zhao X., Wang A., Hu X. (2024). Association between insulin receptor substrate 1 gene polymorphism rs1801278 and gestational diabetes mellitus: An updated meta- analysis. Diabetol. Metab. Syndr..

[69] Kahn S.E., Cooper M.E., Del Prato S. (2014). Pathophysiology and treatment of type 2 diabetes: Perspectives on the past, present, and future. Lancet.

[70] White M.F. (2002). IRS proteins and the common path to diabetes. Am. J. Physiol. Endocrinol. Metab..

[71] Tang C.-Y., Man X.-F., Guo Y., Tang H.-N., Tang J., Zhou C.-L., Tan S.-W., Wang M., Zhou H.-D. (2017). IRS-2 Partially Compensates for the Insulin Signal Defects in IRS-1(-/-) Mice Mediated by miR-33. Mol. Cells.

[72] Martin A.R., Kanai M., Kamatani Y., Okada Y., Neale B.M., Daly M.J. (2019). Clinical use of current polygenic risk scores may exacerbate health disparities. Nat. Genet..

[73] Hartling L., Dryden D.M., Guthrie A., Muise M., Vandermeer B., Donovan L. (2013). Benefits and harms of treating gestational diabetes mellitus: A systematic review and meta-analysis for the U.S. Preventive Services Task Force and the National Institutes of Health Office of Medical Applications of Research. Ann. Intern. Med..

[74] World Health Organization (1999). Definition, Diagnosis and Classification of Diabetes Mellitus and Its Complications: Report of a WHO Consultation. Part 1, Diagnosis and Classification of Diabetes Mellitus.

[75] Barker D.J., Eriksson J.G., Forsen T., Osmond C. (2002). Fetal origins of adult disease: Strength of effects and biological basis. Int. J. Epidemiol..

[76] Ferrara A., Hedderson M.M., Brown S.D., Albright C.L., Ehrlich S.F., Tsai A.-L., Caan B.J., Sternfeld B., Gordon N.P., Schmittdiel J.A. (2016). The Comparative Effectiveness of Diabetes Prevention Strategies to Reduce Postpartum Weight Retention in Women With Gestational Diabetes Mellitus: The Gestational Diabetes’ Effects on Moms (GEM) Cluster Randomized Controlled Trial. Diabetes Care.

[77] Koivusalo S.B., Rönö K., Stach-Lempinen B., Eriksson J.G. (2016). Gestational Diabetes Mellitus Can Be Prevented by Lifestyle Intervention: The Finnish Gestational Diabetes Prevention Study (RADIEL): A Randomized Controlled Trial. Diabetes Care.

[78] Shepherd E., Gomersall J.C., Tieu J., Han S., Crowther C.A., Middleton P. (2017). Combined diet and exercise interventions for preventing gestational diabetes mellitus. Cochrane Database Syst. Rev..

[79] Huvinen E., Lahti J., Klemetti M.M., Bergman P.H., Räikkönen K., Orho-Melander M., Laivuori H., Koivusalo S.B. (2022). Genetic risk of type 2 diabetes modifies the effects of a lifestyle intervention aimed at the prevention of gestational and postpartum diabetes. Diabetologia.

[80] Jaaskelainen T., Klemetti M.M. (2022). Genetic Risk Factors and Gene-Lifestyle Interactions in Gestational Diabetes. Nutrients.

[81] Lamri A., Mao S., Desai D., Gupta M., Paré G., Anand S.S. (2020). Fine-tuning of Genome-Wide Polygenic Risk Scores and Prediction of Gestational Diabetes in South Asian Women. Sci. Rep..

[82] Heijmans B.T., Tobi E.W., Stein A.D., Putter H., Blauw G.J., Susser E.S., Slagboom P.E., Lumey L.H. (2008). Persistent epigenetic differences associated with prenatal exposure to famine in humans. Proc. Natl. Acad. Sci. USA.

[83] Wu H., Zhang Y. (2014). Reversing DNA methylation: Mechanisms, genomics, and biological functions. Cell.

[84] Crusell M.K.W., Hansen T.H., Nielsen T., Allin K.H., Rühlemann M.C., Damm P., Vestergaard H., Rørbye C., Jørgensen N.R., Christiansen O.B. (2018). Gestational diabetes is associated with change in the gut microbiota composition in third trimester of pregnancy and postpartum. Microbiome.

[85] Koren O., Goodrich J.K., Cullender T.C., Spor A., Laitinen K., Bäckhed H.K., Gonzalez A., Werner J.J., Angenent L.T., Knight R. (2012). Host remodeling of the gut microbiome and metabolic changes during pregnancy. Cell.

[86] Zeevi D., Korem T., Zmora N., Israeli D., Rothschild D., Weinberger A., Ben-Yacov O., Lador D., Avnit-Sagi T., Lotan-Pompan M. (2015). Personalized Nutrition by Prediction of Glycemic Responses. Cell.

